# Cross-Modal Correspondences Between Temperature and Taste Attributes

**DOI:** 10.3389/fpsyg.2020.571852

**Published:** 2020-09-25

**Authors:** Kosuke Motoki, Toshiki Saito, Rui Nouchi, Motoaki Sugiura

**Affiliations:** ^1^Department of Food Science and Business, Miyagi University, Sendai, Japan; ^2^Institute of Development, Aging and Cancer, Tohoku University, Sendai, Japan; ^3^Japan Society for the Promotion of Science, Tokyo, Japan; ^4^Smart Aging Research Center, Tohoku University, Sendai, Japan; ^5^International Research Institute of Disaster Science, Tohoku University, Sendai, Japan

**Keywords:** temperature, physical warmth, cross-modal correspondence, multisensory experiences, beverages

## Abstract

Temperature is an important characteristic of food and drink. In addition to food-intrinsic temperature (i.e., serving temperature), consumers often experience food-extrinsic temperature (e.g., physical warmth). Emerging research on cross-modal correspondence has revealed that people reliably associate temperature with other sensory features. Building on the literature on cross-modal correspondence and sensation transference theory, the present study aimed to reveal mental representations of temperature–taste correspondence and cross-modal mental representations influencing corresponding sensory/hedonic perceptions of beverages, with a focus on manipulating food-extrinsic warmth. To reveal mental representations of temperature–taste correspondence, Experiment 1 investigated whether temperature words (*warm, cool*) are associated with sensory/hedonic attributes (e.g., sweet, sour, salty, bitter). The results of Experiment 1 demonstrated that *warm* (vs. *cool*) was matched more with saltiness, tastiness, healthfulness, and preference (intention to buy), whereas *cool* (vs. *warm*) was matched more with sourness and freshness. Experiment 2 assessed whether cross-modal mental representations influence corresponding sensory/hedonic perceptions of beverages. The participants wore hot and cold pads and rated sensory/hedonic attributes of Japanese tea (Experiment 2a) or black coffee (Experiment 2b) before and after tasting it. The results of Experiment 2a demonstrated that physical warmth (vs. coldness) increased healthfulness and the intention to buy Japanese tea. The results of Experiment 2b did not reveal any effects of physical warmth on sensory/hedonic ratings. These findings provide evidence of taste–temperature correspondence and provide preliminary support for the influence of food-extrinsic warmth on taste attributes related to positivity.

## Introduction

### Temperature and Food

Temperature is an important characteristic of food and drink. Food-intrinsic temperatures (i.e., serving temperature) as well as food-extrinsic temperatures (e.g., physical or ambient room temperatures) influence judgment and behaviors (McBurney et al., 1973; Moskowitz, 1973; Bartoshuk et al., 1982; Green and Frankmann, 1987, 1988; Cruz and Green, 2000; Schiffman et al., 2000; Engelen et al., 2003; Mony et al., 2013; Kim et al., 2015; Stokes et al., 2016; Pramudya and Seo, 2017, 2018). Food-intrinsic temperatures influence consumer acceptance (Cardello and Maller, 1982; Lee and O’Mahony, 2002; Brown and Diller, 2008). Food and drinks are more acceptable when served in the temperature range at which they are usually consumed (Cardello and Maller, 1982). In addition, food-extrinsic temperatures are commonly involved in dining settings. People sometimes warm themselves with a gas/electric heater and/or by wearing warm clothes as well as cool themselves with a ceiling fan and/or by wearing light clothes. Moreover, food-extrinsic temperatures influence consumer preferences (Zwebner et al., 2014; Motoki et al., 2018b). For example, physical warmth increases one’s willingness to pay for food (Zwebner et al., 2014). Taken together, this evidence suggests that temperature, even in the food-extrinsic form, influences consumer preference.

### Temperature-Cased Cross-Modal Correspondence

A recent study reviewed temperature-based cross-modal correspondence (Spence, 2020a). Cross-modal correspondence refers to sensory interactions among the senses (Spence, 2011). People map features in one sensory modality onto features in other modalities in a surprisingly consistent manner (e.g., sweet and round shape or high pitch) (e.g., Velasco et al., 2016; Motoki et al., 2019a,c). Previous research has documented correspondence between temperature and the other sensory modalities (Berry, 1961; Wright, 1962; Hardin, 2000; Michael and Rolhion, 2008; Velasco et al., 2013; Ho et al., 2014a,b; Lorentz et al., 2016; Wang and Spence, 2017; Wnuk et al., 2017; Roque et al., 2018; Motoki et al., 2019b). For example, warm (vs. cold) temperatures are perceived to be well matched with visual features, such as a red hue (Ho et al., 2014b) or light color (Motoki et al., 2019b), whereas visual features of coldness (e.g., ice cubes) facilitate categorizing a drink as fresh (Roque et al., 2018). In addition, temperatures are associated with different sound properties (Velasco et al., 2013; Wang and Spence, 2017). Warm (vs. cold) temperatures of beverages are likely to be associated with a lower pitch and a slower tempo (Wang and Spence, 2017). However, most previous research has investigated visual–temperature or sound–temperature correspondence (but see Wnuk et al., 2017). Although temperature is involved in various dining settings (Velasco et al., 2013; Zwebner et al., 2014; Kim et al., 2015; Motoki et al., 2018b), no study has investigated temperature–taste correspondence.

Some research on cross-modal correspondence is based on mental representations without actual experiences of sensory stimuli. Word matching has been used to investigate mental representations of temperature-based or taste-based correspondence (e.g., Velasco et al., 2015; Motoki et al., 2019b) because previous research suggests that cross-modal correspondence may result even when sensing words are used without sensory experiences (Spence et al., 2015; Velasco et al., 2015, 2018b; Saluja and Stevenson, 2018). For example, words related to sweetness/bitterness are well matched with roundness/angularity, and sweet/bitter tastants are similarly well matched with roundness/angularity (Velasco et al., 2015). Our first aim in this study was to reveal mental representations of temperature–taste correspondence and how people associate temperature words with taste attributes.

### Sensation Transference Theory

Food-extrinsic temperatures (e.g., positive experiences of physical warmth) can transfer to corresponding sensory/hedonic ratings (e.g., positive evaluations of food products). Sensation transference theory suggests that food-extrinsic somesthetic sensory experiences can be transferred to expectations and experiences around food and drink (Cheskin, 1972). For example, food-extrinsic haptic sensations (e.g., the weight of cutlery) influence corresponding expectations and experiences of food and beverages (e.g., the perceived texture of foods) (Krishna and Morrin, 2008; Mobini et al., 2011; Piqueras-Fiszman and Spence, 2012; Tu et al., 2015; Biggs et al., 2016; Slocombe et al., 2016; Kampfer et al., 2017; Wang and Spence, 2018). In addition, cross-modal mental representations observed in word matching affect the corresponding taste experience. The words *sweetness/sourness* are well matched with round/angular typefaces, and foods (jelly beans) rated as sweeter and less sour are associated with round (vs. angular) typefaces (Velasco et al., 2018b). Thus, findings of cross-modal mental representations by means of word matching may be reflected in corresponding taste perceptions. The second aim of this study was to reveal whether cross-modal mental representations affect corresponding sensory/hedonic perceptions of beverages, with a focus on manipulating food-extrinsic warmth.

### Hypotheses

We established our hypotheses based on the emotion mediation hypothesis of cross-modal correspondence. The emotion mediation hypothesis suggests that people associate sensory attributes with other attributes based on similarity in emotional meaning (Spence, 2020a). For example, roundness and sweet taste are well matched because both have a positive valence relative to angularity and other tastes (Velasco et al., 2015). Previous studies imply that warm temperatures have a positive valence (Stokes et al., 2016; Pramudya and Seo, 2017). For example, coffee and green tea served at higher temperatures are evaluated positively (e.g., happy and satisfied), whereas beverages served at lower temperatures are evaluated negatively (e.g., disgusting and bitter) (Pramudya and Seo, 2017). In addition, experiences of physical warmth induce positive emotions and lead to greater intentions to buy products (Zwebner et al., 2014). Thus, the word *warmth* (vs. *coolness*) would be more associated with positive taste terms (e.g., tastiness, healthfulness, buying intention, and sweetness), and experiences of physical warmth (vs. coldness) increase ratings of positive taste attributes (e.g., tastiness, healthfulness, buying intention, and sweetness). By contrast, the word *coolness* (vs. *warmth*) is more associated with negativity (e.g., bitterness), and experiences of physical warmth (vs. coldness) increase ratings of negative taste attributes (e.g., bitterness).

H1a: The word *warmth* (vs. *coolness*) is more associated with taste terms linked to positivity (e.g., tastiness, healthfulness, buying intention, and sweetness).

H1b: The word *coolness* (vs. *warmth*) is more associated with taste terms linked to negativity (e.g., bitterness).

H2a: Physical warmth (vs. coldness) increases ratings of positive taste attributes (e.g., tastiness, healthfulness, buying intention, and sweetness).

H2b: Physical coldness (vs. warmth) increases ratings of negative taste attributes (e.g., bitterness).

### The Current Study

Building on the literature on cross-modal correspondence and sensation transference theory, the present study aimed to reveal temperature–taste correspondence. This study investigated mental representations of temperature–taste correspondence and whether cross-modal mental representations influence corresponding sensory/hedonic perceptions of beverages, with a focus on manipulating food-extrinsic warmth. In Experiment 1, participants were asked to determine how much temperature words (*warm, cool*) are associated with sensory/hedonic attributes (e.g., sweet, sour, salty, bitter). In Experiment 2, participants who physically experienced warm or cold temperatures were asked to rank sensory/hedonic attributes of Japanese tea (Experiment 2a) and black coffee (Experiment 2b) before and after tasting each beverage.

## Experiment 1

### Materials and Methods

#### Participants

A total of 103 participants (32 females and 69 males; two participants did not reveal their gender; age: 42.06 ± 9.45 years) gave their online informed consent to take part in the experiment. The sample size was based on recent online research on taste-based correspondence (Velasco et al., 2018a; Motoki et al., 2019d). The participants were recruited on Lancers^1^ and completed the survey on Qualtrics^2^. The platforms allowed the participants to receive monetary compensation for completing the study (50 JPY). This research was conducted in September in Japan. All procedures were conducted according to the Declaration of Helsinki. The experimental protocol was approved by the Ethics Committee of Tohoku University School of Medicine.

#### Procedure

**FIGURE 1:**
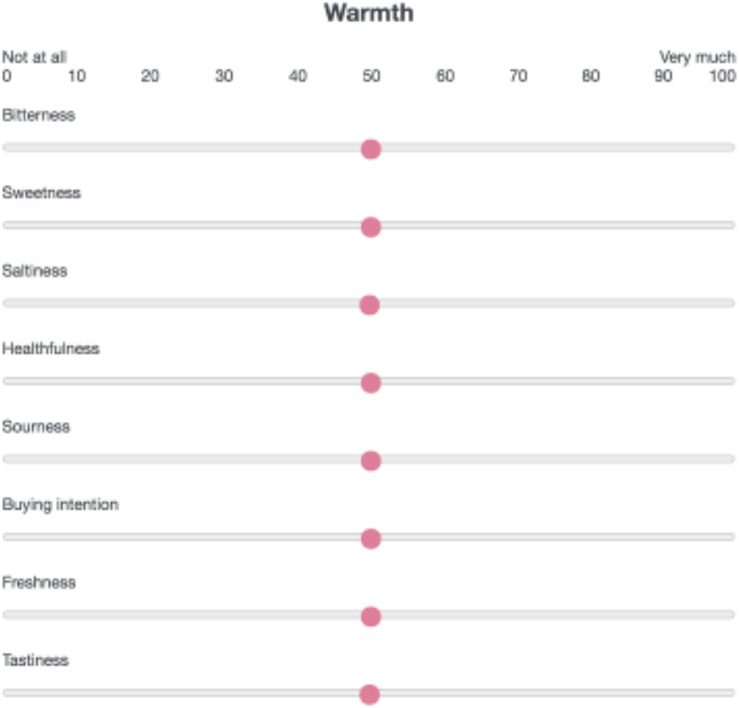
Temperature–taste matching task. Participants were asked to match a temperature word (warmth, coolness) with a sensory/hedonic attribute (tastiness, healthfulness, sweetness, sourness, saltiness, bitterness, freshness, buying intention) using a visual analog scale ranging from 0 (not at all) to 100 (very much).

The study used a within-participants design, with sensory/hedonic attribute (tastiness, healthfulness, sweetness, sourness, saltiness, bitterness, freshness, and buying intention) as the dependent variables and temperature (warm, cool) as the independent variable. Participants were informed that this study aimed to investigate how people associate warmth/coolness with taste attributes. Participants were asked to match temperature words (*warmth* and *coolness*) with each sensory/hedonic attribute (Figure 1). Buying intention was taken as an indicator of consumer preference. This measure was also used in Experiment 2. In total, there were 16 trials in which participants matched each word with a value on a visual analog scale (VAS) ranging from 0 (not at all) to 100 (very much). The order of the trials and rating attributes were randomized across participants.

#### Statistical Analyses

We calculated the degree to which the warm and cold words were matched with taste attributes using paired t tests. *p* < 0.006 (0.05/8) was considered significant with the Bonferroni correction. This procedure was similar to that used in a previous study on cross-modal correspondence (Motoki et al., 2019b).

We also conducted multivariate analysis of covariance (MANCOVA) to assess the effects of temperature (warm, cool) on taste ratings (tastiness, healthfulness, sweetness, sourness, saltiness, bitterness, warmth, freshness, and buying intention), with gender as a covariate. Gender was included because a previous study showed gender differences in perceptions of thermal sensation (Karjalainen, 2007). Two participants did not reveal their gender, so those two participants were excluded from analyses. The final sample included 101 participants. Each sensory/hedonic attribute was rated on a scale from 1 to 7. We focused on the interactive effects of temperature and gender. Data were analyzed with SPSS software (version 25.0; SPSS Inc., Chicago, IL, United States).

### Results

**FIGURE 2:**
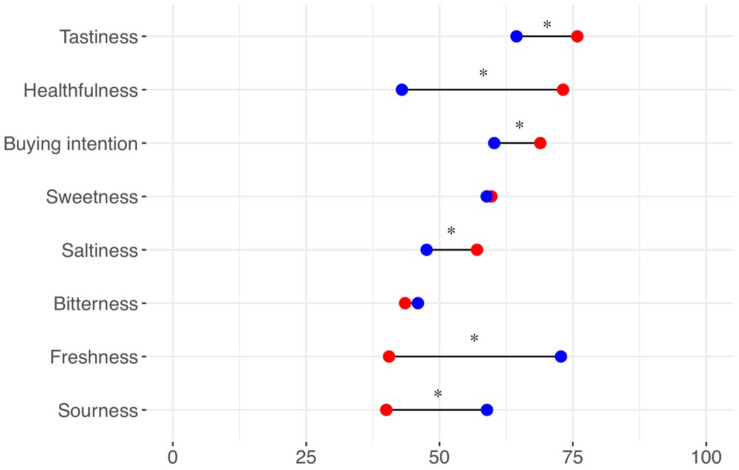
An illustration of temperature–taste attribute matching. The match between temperature words (warmth, coolness) and sensory/hedonic attributes was rated using a scale ranging from 0 (not at all) to 100 (very much). Red circles represent the mean data for warmth. Blue circles represent the mean data for coolness. An asterisk indicates a significant difference at *p* < 0.006 (0.05/8).

The word *warm* was matched more often with saltiness, tastiness, healthfulness, and buying intention than the word *cool*. By contrast, the word *cool* was matched more often with sourness and freshness than the word *warm* (Figure 2). The words *warm* and *cool* did not differ in terms of matching with sweetness and bitterness. A statistical summary is shown in Table 1.

**TABLE 1:** Temperature–taste attribute matching.

	**Warmth**	**Coolness**	***p*-value**	**Cohen *d***
Buying intention	68.89 (15.72)	60.26 (19.43)	< 0.001	0.364
Tastiness	75.83 (14.01)	64.43 (17.72)	< 0.001	0.554
Healthfulness	73.17 (14.67)	42.94 (18.30)	< 0.001	1.160
Sweetness	59.72 (19.76)	58.85 (22.58)	0.769	0.029
Sourness	40.02 (21.99)	58.90 (23.17)	< 0.001	–0.581
Saltiness	57.03 (20.93)	47.58 (20.66)	< 0.001	0.375
Bitterness	43.54 (19.35)	45.95 (23.10)	0.400	–0.083
Freshness	40.54 (21.26)	72.77 (18.58)	< 0.001	–0.989

*Mean (± SD) data for the match between temperature words (warmth, coldness) and sensory/hedonic attributes, rated using a scale ranging from 0 (not at all) to 100 (very much); df = 102.*

The MANCOVA results revealed a significant main effect of temperature [*F*_(8, 92)_ = 28.967, Wilks’ lambda = 0.284, *p* < 0.001, η*_*p*_*^2^ = 0.716] and an interaction effect of temperature and gender [*F*_(8, 92)_ = 2.395, Wilks’ lambda = 0.828, *p* = 0.022, η*_*p*_*^2^ = 0.172]. No main effect of gender was observed [*F*_(8, 92)_ = 1.133, Wilks’ lambda = 0.910, *p* = 0.349, η*_*p*_*^2^ = 0.090]. Univariate ANCOVA showed that the word *cool* matched more frequently with saltiness and freshness than *warm.* A further univariate ANCOVA revealed significant interactive effects of temperature and gender on sour, healthfulness and buying intention. The matching scores for warmth–healthfulness [*F*_(1, 99)_ = 6.333, *p* = 0.014, η^2^*_*p*_* = 0.060] and warmth–buying intention [*F*_(1, 99)_ = 5.973, *p* = 0.016, η^2^*_*p*_* = 0.057] were higher in females than in males. In contrast, no differences were observed between males and females in coolness–healthfulness [*F*_(1, 99)_ = 1.503, *p* = 0.223, η^2^*_*p*_* = 0.015] or coolness–buying intention [*F*_(1, 99)_ = 0.670, *p* = 0.415, η^2^*_*p*_* = 0.007]. The matching score of coolness–sourness [*F*_(1, 99)_ = 8.969, *p* = 0.004, η^2^*_*p*_* = 0.083] was higher in males than in females. By contrast, no differences were observed between males and females in warmth–sourness [*F*_(1, 99)_ = 0.561, *p* = 0.456, η^2^*_*p*_* = 0.006]. A statistical summary is shown in Table 2.

**TABLE 2:** Results of univariate analysis of covariance investigating the effects of temperature and gender on taste ratings.

**Effect**	**Rating type**	***F***	***p*-value**	**η*_*p*_*^2^**
Temperature	Buying intention	0.856	0.357	0.009
	Tastiness	0.672	0.414	0.007
	Healthfulness	3.461	0.066	0.034
	Sweetness	2.85	0.095	0.028
	Sourness	0.127	0.723	0.001
	Saltiness	7.072	0.009	0.067
	Bitterness	1.083	0.301	0.011
	Freshness	8.008	0.006	0.075
Temperature × gender	Buying intention	5.306	0.023	0.051
	Tastiness	1.335	0.251	0.013
	Healthfulness	5.136	0.026	0.049
	Sweetness	3.468	0.066	0.034
	Sourness	6.664	0.011	0.063
	Saltiness	2.184	0.143	0.022
	Bitterness	2.037	0.157	0.020
	Freshness	0.322	0.572	0.003

*The model included temperature (warm and cool) as a within-participants factor and gender as a between-participants factor; df = 99.*

The results of Experiment 1 revealed that people have specific associations between temperatures and sensory/hedonic attributes. Experiment 2 investigated whether these associations have consequences for taste expectations and experiences.

## Experiment 2

The aim of Experiment 2 was to reveal whether cross-modal mental representations (the findings of Experiment 1) affect corresponding sensory/hedonic perceptions of beverages, with a focus on manipulating food-extrinsic warmth. We investigated how physical warmth influences corresponding sensory/hedonic ratings of Japanese tea (Experiment 2a) and black coffee (Experiment 2b). These two beverages were chosen because both are popular beverages in Japan (Pitelka, 2013; Whitelaw, 2014) and therefore might be consumed in situations involving food-extrinsic temperatures.

### Materials and Methods

#### Design and Participants

The experimental design was a 2 (physical warmth: warm, cold) × 2 (time: pretasting, posttasting) design in which all factors were within-participants variables. A total of 69 participants gave their informed consent to take part in one of the two experiments. Data from 35 participants were included in the final analyses for Experiment 2a (seven females; age: 20.77 ± 1.48 years), and data from 34 participants were included in the final analyses for Experiment 2b (15 females; age: 21.41 ± 3.21 years). The relatively small sample sizes were due to time constraints in data collection. The participants were recruited through notices on a university campus and provided written informed consent after receiving an explanation of the experiment. All procedures were conducted according to the Declaration of Helsinki. The experimental protocol was approved by the Ethics Committee of Tohoku University School of Medicine.

#### Stimuli

We used different beverages in Experiments 2a and 2b to test for the generalizability of the temperature effects on sensory/hedonic perceptions of beverages. In Experiment 2a, Japanese tea (Oi Ocha Roasted rice tea with matcha; Itoen, Tokyo, Japan) was used^3^. The ingredients of the Japanese tea included roasted rice, tea leaves, matcha, and vitamin C. In Experiment 2b, black coffee (Nescafé Gold blend full-boiled; Nestle, Tokyo, Japan) was used^4^. The black coffee was decaffeinated and served without sugar. The bottled Japanese tea (Experiment 2a) or black coffee (Experiment 2b) was poured into a white paper cup (about 10 mL per cup) and served to the participants. Both the Japanese tea and black coffee were stored at room temperature, and the serving temperature was not assessed in either experiment. Previous studies used about 10 mL of each solution and confirmed that this was sufficient for participants to perceive different taste qualities (Crisinel and Spence, 2011; Wang et al., 2016). Both Japanese tea and black coffee were stored at room temperature. As in previous studies (Crisinel and Spence, 2011; Wang et al., 2016), the serving temperature was not recorded because both beverages were stored in the same room in the second and third week of March. Thus, it is unlikely that the temperatures of the beverages varied across participants.

#### Manipulation of Physical Warmth

**FIGURE 3:**
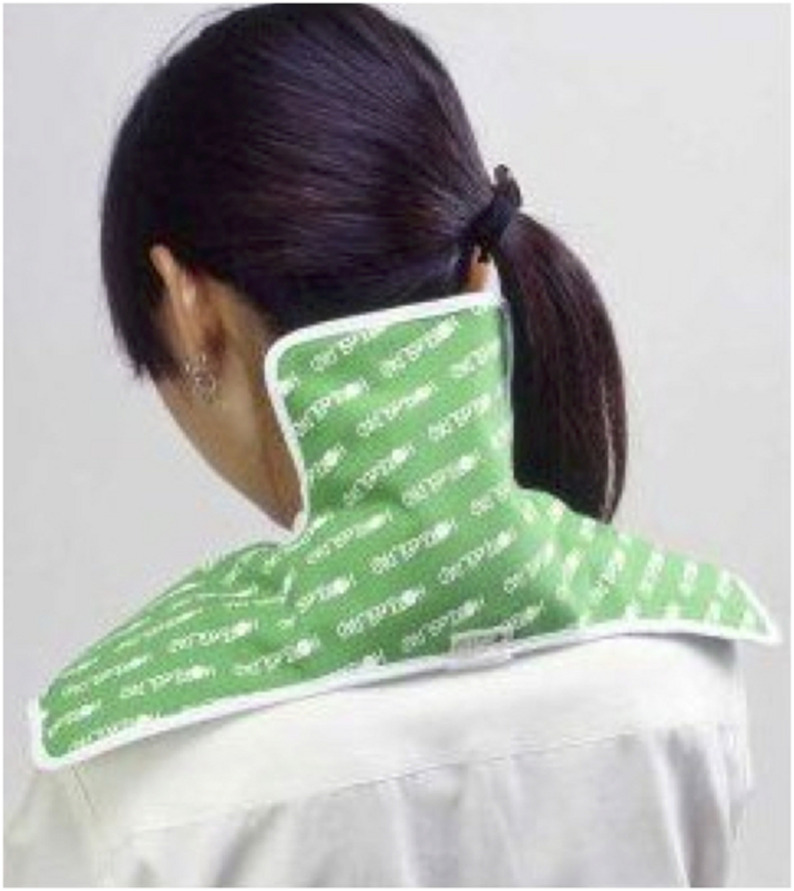
Hot/cold pad.

Participants wore a hot (warm condition) or a cold (cold condition) pad (Figure 3) around their neck. The warm and cold pads were of the same design. The procedure followed that of a previous study featuring the manipulation of physical warmth (Motoki et al., 2019b). It has been shown that this manipulation significantly affects subjective thermal sensations (Motoki et al., 2019b). The hot and cold pads were used following the manufacturer’s instruction and were about 50°C and −5°C, respectively (Motoki et al., 2019b).

#### Procedure

**FIGURE 4:**
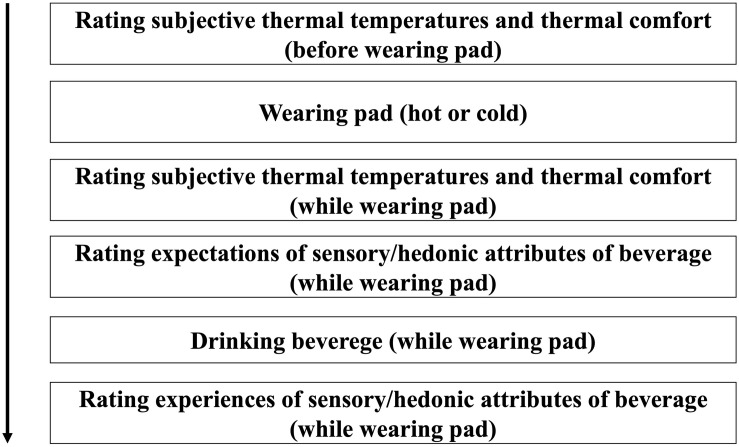
Timeline for the experimental procedure. The procedure was repeated in the other condition (hot or cold), and the order of the conditions was randomly allocated.

Participants (one at a time) were led to a table and sat in a chair. At the start of the experiments, participants were told that they would drink tea (Experiment 2a) or coffee (Experiment 2b) while wearing a hot/cold pad. The timeline of the experimental procedure is shown in Figure 4.

The two drinks were served at the same temperature, although we told the participants that both drinks were different. Before they put on the pad (hot or cold), they answered baseline questions on thermal temperature (1: very cold to 7: very hot) and thermal comfort (1: very negative to 7: very positive) using a 7-point Likert scale. After putting on the pad (hot or cold), participants answered the temperature and comfort questions again.

While wearing the pad, participants saw the served drinks (Japanese tea for Experiment 2a and black coffee for Experiment 2b) and gave their expected sensory/hedonic ratings (tastiness, healthfulness, sweetness, sourness, saltiness, bitterness, drink warmth, freshness, buying intention). The rating procedure used a 7-point Likert scale ranging from 1 (not at all) to 7 (very much) for each question. After participants provided their expected ratings, they drank the beverage and provided their experience ratings (tastiness, healthfulness, sweetness, sourness, saltiness, bitterness, drink warmth, freshness, and buying intention). The procedure was repeated in the other condition (hot or cold), and the order of the conditions was randomly allocated. The participants rested for about 1 min between conditions. None of the participants in either of the two experiments were informed about the brand name, origin, taste, temperature, or flavor profile of the drinks that they were served; they only knew that they would drink Japanese tea (Experiment 2a) and coffee (Experiment 2b). To reduce discomfort during wearing the pads, it took a short amount of time (about 30–60 s) to complete the thermal and beverage ratings. The time required to complete the entire procedure was about 5 min.

#### Statistical Analyses

To verify the influence of physical warmth on thermal sensation and comfort, we conducted paired *t*-tests on the change in scores (while wearing the hot/cold pad minus before wearing the pad).

For both Experiments 2a and 2b, repeated-measures (RM) ANCOVAs were conducted to assess the effects of physical warmth (warm, cold) and time (pretasting, posttasting) on the taste ratings (tastiness, healthfulness, sweetness, sourness, saltiness, bitterness, warmth, freshness, and buying intention) with gender and thermal comfort as covariates. Gender was included as a covariate because a previous study reported gender differences in perceptions of thermal sensation (Karjalainen, 2007), and the results of Experiment 1 showed gender differences. Each sensory/hedonic attribute rated on a scale from 1 to 7 was a dependent variable in the RM-ANCOVAs. The analyses were conducted with SPSS version 25 (IBM, Chicago, IL, United States).

#### Results of Manipulating Physical Warmth

The means and SDs of thermal sensation were wearing the hot pad (Experiment 2a: *M* = 5.600 ± 0.695; Experiment 2b: *M* = 5.382 ± 0.922), before wearing the hot pad (Experiment 2a: *M* = 2.914 ± 0.818; Experiment 2b = 3.029 ± 0.797), wearing the cold pad (Experiment 2a: *M* = 1.714 ± 0.667; Experiment 2b: *M* = 1.971 ± 0.717), and before wearing the cold pad (Experiment 2a: *M* = 3.171 ± 0.857; Experiment 2b = 3.353 ± 0.883). The means and SDs of thermal comfort were wearing the hot pad (Experiment 2a: *M* = 5.551 ± 1.222; Experiment 2b: *M* = 5.529 ± 1.134), before wearing the hot pad (Experiment 2a: *M* = 3.543 ± 1.197; Experiment 2b = 4.147 ± 1.282), wearing the cold pad (Experiment 2a: M = 2.686 ± 1.255; Experiment 2b: M = 3.235 ± 1.281), and before wearing the cold pad (Experiment 2a: *M* = 4.086 ± 1.337; Experiment 2b = 4.588 ± 1.373).

Thermal sensation in the warm condition was rated as warmer than that in the cold condition (Experiment 2a: M_hot pad–before hot pad_ = 2.686 ± 0.832 vs. M _cold pad–before cold pad_ = –1.457 ± 0.950, *t*_34_ = 21.977, *p* < 0.001; Experiment 2b: M_hot pad–before hot pad_ = 2.353 ± 1.252 vs. M_cold pad–before cold pad_ = –1.382 ± 0.817, *t*_33_ = 16.930, *p* < 0.001). Thermal comfort in the warm condition was rated more favorably than that in the cold condition (Experiment 2a: M_hot pad–before hot pad_ = 1.971 ± 1.689 vs. M_cold pad–before cold pad_ = –1.400 ± 0.976, *t*_34_ = 10.699, *p* < 0.001; Experiment 2b: M_hot pad–before hot pad_ = 1.382 ± 1.701 vs. M_cold pad–before cold pad_ = –1.353 ± 1.276, *t*_33_ = 8.958, *p* < 0.001). Although the thermal sensation manipulations were successful, a difference in thermal comfort was observed between the warm and cold conditions. Thus, we included a thermal comfort score (changed comfort in the warm condition – changed comfort in the cold condition) as a covariate in further analyses.

#### Results for Japanese Tea (Experiment 2a)

**FIGURE 5:**
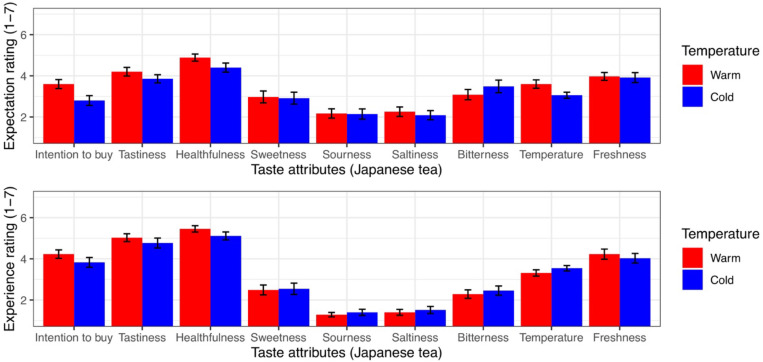
The influence of physical temperature on the taste attributes of Japanese tea. The error bars represent standard error.

The analyses revealed significant main effects of physical warmth and time. However, no main effects of gender or thermal comfort or any interactive effects were observed (*p* > 0.05; Table 3). Univariate ANCOVAs showed that the participants in the warm (vs. cold) temperature increased their buying intention and healthfulness ratings (Table 4). Further univariate ANCOVAs showed that posttasting (vs. pretasting) increased the buying intention, tastiness, and healthfulness ratings but decreased ratings of sourness, saltiness, and bitterness (Appendix Table A). The statistics are shown in Table 5. The results are illustrated graphically in Figure 5. Note that the main results were unchanged when we ran ANOVAs without the covariates (gender and thermal comfort).

**TABLE 3:** Results of repeated-measures analyses of covariance investigating the effects of temperature (warm and cold) and time (pretasting and posttasting) on taste ratings with gender and thermal comfort as covariates.

**Effect**	***F***	**Wilks’ lambda**	***p-*value**	**η*_*p*_*^2^**
Temperature	2.672	0.500	0.026	0.500
Time	11.106	0.194	< 0.001	0.806
Temperature × time	1.202	0.689	0.339	0.331
Temperature × thermal comfort	1.525	0.636	0.196	0.364
Temperature × gender	0.910	0.746	0.532	0.254
Time × thermal comfort	1.901	0.584	0.101	0.416
Time × gender	1.202	0.689	0.338	0.311
Temperature × time × thermal comfort	0.813	0.766	0.610	0.234
Temperature × time × gender	0.869	0.754	0.565	0.246

**TABLE 4:** Results of univariate analyses of covariance investigating the effects of temperature on taste ratings.

**Rating type**	***F***	***p*-value**	**η*_*p*_*^2^**
Buying intention	18.48	< 0.001	0.37
Tastiness	2.00	0.167	0.06
Healthfulness	5.66	0.002	0.27
Sweetness	0.41	0.529	0.01
Sourness	0.50	0.483	0.02
Saltiness	0.26	0.612	0.01
Bitterness	1.45	0.237	0.04
Temperature	0.27	0.605	0.01
Freshness	0.98	0.330	0.03

**The design included temperature (warm and cold) and time (pretasting and posttasting) as within-participants factors and gender and thermal comfort as covariates.**

**TABLE 5:** Basic statistical summary of Experiment 2a.

**Rating type**	**Expectation**		**Experience**	
	**Warm**	**Cold**	**Warm**	**Cold**
Buying intention	3.60 (1.29)	2.80 (1.41)	4.23 (1.22)	3.83 (1.40)
Tastiness	4.20 (1.23)	3.86 (1.17)	5.03 (1.12)	4.77 (1.42)
Healthfulness	4.89 (1.02)	4.40 (1.31)	5.46 (1.12)	5.11 (1.13)
Sweetness	2.97 (1.71)	2.91 (1.72)	2.49 (1.42)	2.54 (1.63)
Sourness	2.17 (1.34)	2.14 (1.48)	1.29 (0.62)	1.40 (0.98)
Saltiness	2.26 (1.36)	2.09 (1.31)	1.40 (0.85)	1.51 (1.01)
Bitterness	3.09 (1.48)	3.49 (1.81)	2.29 (1.23)	2.46 (1.34)
Temperature	3.60 (1.19)	3.06 (0.87)	3.31 (0.90)	3.54 (0.78)
Freshness	3.97 (1.12)	3.91 (1.42)	4.23 (1.46)	4.03 (1.38)

*The influence of physical warmth on expectations and experiences of Japanese tea on a 7-point Likert scale ranging from 1 (not at all) to 7 (very much) for each rating. Standard deviations are in parentheses.*

#### Results for Black Coffee (Experiment 2b)

**FIGURE 6:**
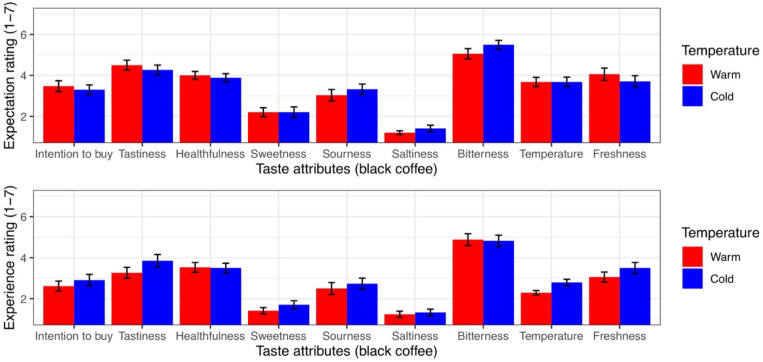
The influence of physical temperature on the taste attributes of black coffee. The error bars represent standard error.

The analyses revealed significant main effects of time. However, no main effects of temperature, gender, or thermal comfort or any interactive effects were observed (Table 6). Follow-up univariate ANCOVAs revealed that pretasting (vs. posttasting) increased the buying intention, tastiness, healthfulness, sweetness, drink warmth, and freshness ratings (Appendix Table B). The basic statistics are shown in Table 7. The data are illustrated graphically in Figure 6. Note that the main results were unchanged when we ran the ANOVAs without the covariates (gender and thermal comfort).

**TABLE 6:** Results of repeated-measures analyses of covariance investigating the effects of temperature (warm and cold) and time (pretasting and posttasting) on taste ratings with gender and thermal comfort as covariates.

**Effect**	***F***	**Wilks’ lambda**	***p-*value**	**η*_*p*_*^2^**
Temperature	1.160	0.688	0.365	0.312
Time	7.277	0.260	< 0.001	0.740
Temperature × time	1.657	0.607	0.158	0.393
Temperature × thermal comfort	0.797	0.762	0.623	0.238
Temperature × gender	1.391	0.648	0.249	0.352
Time × thermal comfort	1.647	0.608	0.160	0.392
Time × gender	0.842	0.752	0.586	0.248
Temperature × time × thermal comfort	1.534	0.625	0.195	0.375
Temperature × time × gender	0.758	0.771	0.654	0.229

**TABLE 7:** Basic statistical summary of Experiment 2b.

**Rating type**	**Expectation**		**Experience**	
	**Warm**	**Cold**	**Warm**	**Cold**
Buying intention	3.47 (1.54)	3.39 (1.38)	2.62 (1.44)	2.91 (1.62)
Tastiness	4.50 (1.42)	4.26 (1.40)	3.26 (1.56)	3.85 (1.76)
Healthfulness	4.00 (1.10)	3.88 (1.18)	3.53 (1.40)	3.50 (1.35)
Sweetness	2.21 (1.27)	2.21 (1.49)	1.41 (0.89)	1.71 (1.14)
Sourness	3.03 (1.62)	3.32 (1.47)	2.50 (1.69)	2.74 (1.58)
Saltiness	1.21 (0.54)	1.41 (0.93)	1.24 (0.89)	1.32 (0.98)
Bitterness	5.06 (1.46)	5.50 (1.24)	4.88 (1.68)	4.82 (1.60)
Temperature	3.68 (1.32)	3.68 (1.39)	2.29 (0.63)	2.79 (0.91)
Freshness	4.06 (1.76)	3.71 (1.62)	3.06 (1.43)	3.50 (1.58)

*The influence of physical warmth on expectations and experiences of black coffee. Various parameters are rated on a 7-point Likert scale ranging from 1 (not at all) to 7 (very much). Standard deviations are in parentheses.*

### Discussion

This study aimed to determine temperature–taste correspondence. We investigated mental representations of temperature–taste correspondence and cross-modal mental representations that influence corresponding sensory/hedonic perceptions of beverages, with a focus on manipulating the extrinsic warmth of food. The results of Experiment 1 showed that the word *warmth* (vs. *coldness*) was matched more with saltiness, tastiness, healthfulness, and buying intention. By contrast, the word *coldness* (vs. *warmth*) was matched more with sourness and freshness. The results of Experiment 2a revealed that the experience of physical warmth (vs. coldness) increased healthfulness and buying intention for Japanese green tea. The results of Experiment 2b did not reveal any changes in sensory/hedonic attributes of black coffee following warm versus cold temperatures. These findings provide the evidence of taste–temperature correspondence and provide preliminary support for the association between food-extrinsic temperature and corresponding hedonic attributes.

#### Temperature–Taste Correspondence

The results of Experiment 1 revealed that people have specific associations between temperature and sensory/hedonic attributes. Although the emerging literature on cross-modal correspondence has shown that temperature has a specific association with other sensory modalities (Velasco et al., 2013; Ho et al., 2014b; Wang and Spence, 2017; Roque et al., 2018; Motoki et al., 2019b), temperature-based cross-modal correspondence has only been reported for visual and auditory features (e.g., warmth and red color). Although a close relationship has been shown between temperature and taste attributes (McBurney et al., 1973; Moskowitz, 1973; Bartoshuk et al., 1982; Green and Frankmann, 1987, 1988; Cruz and Green, 2000; Schiffman et al., 2000; Engelen et al., 2003; Mony et al., 2013; Kim et al., 2015; Stokes et al., 2016; Pramudya and Seo, 2017, 2018), it has not been shown how temperature is specifically associated with taste attributes in the subjective matching task. Our findings show that the word *warmth* (vs. *coldness*) was matched more with saltiness, tastiness, healthfulness, and buying intention. By contrast, the word *coldness* (vs. *warmth*) was matched more with sourness and freshness. Taken together, these findings provide evidence of temperature–taste correspondence and show how people map the concept of temperature onto sensory/hedonic attributes.

The results of Experiment 2 showed that the experience of physical warmth (vs. coldness) increased healthfulness and buying intention for Japanese green tea. This finding was partially consistent with the results of the word-matching task (Experiment 1), such that warmth (vs. coldness) was more associated with the concepts of healthfulness and buying intention. According to embodiment theory (Barsalou, 2010), abstract psychological concepts (e.g., emotional warmth and positive reactions) are metaphorically grounded in concrete physical experiences (e.g., physical warmth) (Barsalou, 2010). Previous research has demonstrated that physically warm temperatures lead to more positive evaluations of beverages (Zwebner et al., 2014). Although the results of Experiment 1 demonstrated that warmth and coolness are matched more with taste attributes (e.g., warmth with saltiness and coolness with sourness), we did not find any effects of physical warmth on basic taste ratings (sweet, sour, salty, bitter). Taken together, these results indicate that an experience of physical warmth increases the evaluation of abstract food concepts (i.e., healthfulness, buying intention) but does not influence basic taste evaluations.

#### Underlying Mechanisms for Taste–Temperature Correspondence

The explanation behind temperature–taste correspondence is linked to statistical regularity (Spence, 2011). People may be regularly exposed to a certain mapping of temperatures and taste-related information. For example, salty food (e.g., miso soup) is often served at a warm temperature. By contrast, sour and fresh food (e.g., gumi and carbonated drinks) is often served cold. Consumers might often see such associations between temperatures and tastes in daily dining settings. Repeated exposure to the statistical co-occurrence of temperature and taste attributes may influence internalization of the environmental statistics.

An alternative explanation has to do with valence matching (Velasco et al., 2015; Kantono et al., 2016, 2019; Motoki et al., 2019d; Xu et al., 2019). As we showed here, warm sensations elicit positive feelings, which transfer to a higher willingness to pay for products (Zwebner et al., 2014). People generally like warm foods (Pramudya and Seo, 2018). Both healthfulness and tastiness are positively correlated with food preferences (Motoki et al., 2018a). Given the similarity in valence, people tend to associate warmth with likable taste attributes (tastiness, healthfulness, and greater buying intention). Although valence matching often reveals changes in sweet and bitter perceptions (Velasco et al., 2015; Motoki et al., 2019d), we did not find any associations between temperature and sweetness or bitterness in Experiment 1 or 2. The results did not fit our hypotheses, possibly because of individual differences in the valence of sweetness and bitterness. Although sweetness is liked and bitterness is disliked in general, the degree of valence of sweet and bitter foods might be more varied than likable taste attributes (tastiness, healthfulness, and greater buying intention). For example, there are individual differences in how people attribute sweet tastes to a positive valence, and some may attribute sweetness to a negative valence (e.g., guilty feelings) (Kampov-Polevoy et al., 2006). Weaker valence matching may have attenuated the effects of temperature on sweet and bitter taste correspondence. We did not measure core affect (valence and arousal), which could improve understanding of putative emotional transfer. For instance, it is not clear whether thermal comfort corresponds to valence (Spence, 2020a).

Previous research has shown that warm and cold temperatures are associated with red and blue, respectively (Ho et al., 2014b). However, in our experiments, the colors of the hot/cold pad (green) and beverages (black for black coffee and green for Japanese green tea) were not associated with previously reported color temperatures. Thus, it seems that our findings are not attributable to color-temperature correspondence.

#### Differential Effects of Physical Warmth on Hedonic/Sensory Perceptions of Two Beverages

The effects of physical warmth on hedonic/sensory ratings were different between Japanese tea and black coffee, although we did not hypothesize a differential influence of physical warmth between the two beverages. The matching of warmth with healthfulness and perception of Japanese tea as healthier than black coffee might have influenced these results. The results of Experiment 1 showed that warmth (vs. coldness) and healthfulness were matched more frequently than any other attribute pair. Thus, warmth may exert more influence on perceived healthfulness than other attributes. The data from Experiment 2 suggested that Japanese tea is perceived to be healthier than black coffee. This result might explain the differential influence of warmth on the perceptions of Japanese tea and black coffee; warmth might increase the perception of healthfulness only when the drink is already regarded as healthy to some extent (e.g., Japanese tea).

#### Possible Applications of the Findings

Our findings have practical implications for industry. The results for Experiment 2a showed that physical warmth increased perceptions of healthfulness and buying intention. Thus, restaurant staff could recommend Japanese tea to customers wearing warm clothes. This could be especially effective for customers who are interested in a healthy lifestyle. Modifying the ambient temperature of the store to increase physical warmth might also be effective.

#### Limitations and Future Directions

A major limitation of this study is its small sample size (*n* = 35 for Experiment 2a, *n* = 34 for Experiment 2b, within-participants design). We did not conduct a sample size calculation for the experiments because no previous research has investigated the effect of extrinsic temperature on hedonic and sensory ratings. We hypothesized an influence of physical warmth on hedonic/sensory perceptions of the two beverages (Japanese tea and black coffee), but no significant effects were observed in Experiment 2b (black coffee). This might have been because of the small sample sizes in this study. Although we did not find any evidence for the effects of temperature on sensory/hedonic ratings of black coffee, this does not mean that the effects were non-existent. The directions of some of the sensory/hedonic attributes of black coffee were consistent with the hypothesis. For example, the mean freshness rating for black coffee was 3.50 at the cold temperature and 3.06 at the warm temperature. Previous research using relatively small sample sizes and a between-participants design did not find significant differences in sensory transference (Machiels, 2018). To detect possible small effects of physical warmth on sensory/hedonic ratings, further study is needed to replicate the current findings with larger sample sizes.

This study checked for the manipulation of physical warmth using subjective feelings of warmth. Although this procedure has been used previously with extrinsic temperature (Wortman et al., 2014; Zwebner et al., 2014; Motoki et al., 2018b, 2019b; Sinha and Bagchi, 2019), it remains unknown how manipulating warmth affects participants’ actual physical temperature. Further studies should measure changes in participants’ actual physical temperature after wearing a hot/cold pad.

An additional limitation was the possible mismatch between the measured temperature (temperature words) in Experiment 1 and the actual temperature (hot/cold pads) in Experiment 2. Participants in Experiment 1 may have perceived the temperature words to relate to a food item. Although previous research suggests a link between oral and physical experiences (e.g., Biggs et al., 2016), associations between food temperature and hedonic/sensory attributes might differ from those between physical temperature and hedonic/sensory attributes. This could explain the differences between the results of Experiments 1 and 2.

There were also limitations pertaining to the within-participants design. In Experiment 2, participants experienced hot and cold conditions and drank the same beverage. Thus, they may have been aware that temperature was a variable of interest in the study. Further studies should aim to replicate our findings using a between-participants design.

This study manipulated food-extrinsic temperature using physical sensation and a neck pad. However, ambient temperatures can be more easily manipulated by store managers than physical warmth. Ambient temperature influences food preferences (Zwebner et al., 2014; Motoki et al., 2018b). It remains unknown how ambient temperature influences expectations and experiences of food and drink and whether the effects of ambient temperature are similar to those of physical warmth. The interaction between ambient temperature and sample temperature might also have influenced the present results. The drinks were stored at room temperature, where warm (vs. cold) ambient temperatures might differentially influence hot and cold drinks. Warm (vs. cold) ambient temperatures might increase the preference for cold (vs. hot) drinks and accordingly influence ratings of taste attributes associated with pleasantness (e.g., tastiness, healthfulness). Ambient temperature may also have influenced the results of the word association task (Experiment 1). Further studies are needed to determine whether manipulating ambient temperature influences taste-temperature associations, as well as drinking expectations and experiences.

Individual differences in consumption patterns/preferences might also have affected the results. We did not investigate the participants’ tea and coffee consumption patterns or preferences. Some of the participants might habitually drink hot (rather than room temperature) Japanese tea and black coffee. Additionally, personal preference for Japanese tea or black coffee may have influenced the results.

Cultural, demographic and individual factors should also be considered. Consuming (Japanese) green tea and non-hot coffee is considered normal in Japan. Thus, a similar study including participants from other cultures might yield different findings. Additionally, the age range of the participants was different between Experiments 1 and 2. The participants in Experiment 2 were all university students, and were younger than those in Experiment 1. Also, the proportion of male participants was higher in Experiment 2 than Experiment 1. These differences may have contributed to the different results between Experiments 1 and 2. Moreover, we did not collect smell or taste impairment data. Further studies should investigate the generalizability of our findings by manipulating cultural, demographic and individual factors.

## Conclusion

This study investigated temperature–taste correspondence. The word *warm* (vs. *cool*) was matched more frequently with saltiness and positive attributes (tastiness, healthfulness, and buying intention). By contrast, the word *cool* (vs. *warm*) was matched more frequently with sourness and freshness. Further experiments showed that physical warmth (vs. coldness) increased healthfulness and buying intention of Japanese green tea, although no effects of physical warmth on sensory/hedonic perceptions were observed for black coffee. These findings provide evidence of taste–temperature correspondence and provide preliminary support for the influence of food-extrinsic warmth on taste attributes related to positivity.

## Data Availability Statement

The raw data supporting the conclusions of this article will be made available by the authors, without undue reservation, to any qualified researcher.

## Ethics Statement

The studies involving human participants were reviewed and approved by the Ethics Committee of Tohoku University School of Medicine. The patients/participants provided their written informed consent to participate in this study.

## Author Contributions

KM: conceptualization, formal analysis, writing – original draft. KM and TS: data curation. KM and RN: funding acquisition. KM, TS, RN, and MS: investigation, methodology. MS: resources, supervision. TS, RN, and MS: writing – review and editing. All authors contributed to the article and approved the submitted version.

## Conflict of Interest

The authors declare that the research was conducted in the absence of any commercial or financial relationships that could be construed as a potential conflict of interest.

## Footnotes

1 https://www.lancers.jp/

2 https://www.qualtrics.com/jp/

3 http://www.itoen.co.jp/products/detail.php?id=51

4 https://shop.nestle.jp/front/contents/machine/ics/bottle/
