# Supplementary material for: Cross-Modal Correspondences Between Temperature and Taste Attributes
Source: Front Psychol. 2020 Sep 25;11:571852. doi: 10.3389/fpsyg.2020.571852 (PMC7546214; doi:10.3389/fpsyg.2020.571852)
Supplement: Supplementary file 1 [file Table_1.docx]

**Appendix Table A.** Results of analyses of variance investigating the effects of gender on temperature–taste matching in Experiment 1. The design included temperature (warm and cold) as a within-participants factor and gender (male and female) as a between-participants factor.

|  | Effect | *F* | *p-value* | *ηp2* |
| --- | --- | --- | --- | --- |
| Buying intention | Gender | 0.765 | 0.384 | 0.008 |
|  | Temperature | 18.711 | <.001 | 0.159 |
|  | Temperature × Gender | 5.306 | 0.023 | 0.051 |
| Tastiness | Gender | 0.710 | 0.401 | 0.007 |
|  | Temperature | 33.124 | <.001 | 0.251 |
|  | Temperature × Gender | 1.335 | 0.251 | 0.013 |
| Healthfulness | Gender | 0.566 | 0.454 | 0.006 |
|  | Temperature | 143.881 | <.001 | 0.592 |
|  | Temperature × Gender | 5.136 | 0.026 | 0.049 |
| Sweetness | Gender | 4.990 | 0.028 | 0.048 |
|  | Temperature | 0.759 | 0.386 | 0.008 |
|  | Temperature × Gender | 3.468 | 0.066 | 0.034 |
| Sourness | Gender | 2.738 | 0.101 | 0.027 |
|  | Temperature | 45.560 | <.001 | 0.315 |
|  | Temperature × Gender | 6.664 | 0.011 | 0.063 |
| Saltiness | Gender | 4.068 | 0.046 | 0.040 |
|  | Temperature | 8.967 | 0.004 | 0.083 |
|  | Temperature × Gender | 2.184 | 0.143 | 0.022 |
| Bitterness | Gender | 0.052 | 0.819 | 0.001 |
|  | Temperature | 1.893 | 0.172 | 0.019 |
|  | Temperature × Gender | 2.037 | 0.157 | 0.020 |
| Freshness | Gender | 4.618 | 0.034 | 0.045 |
|  | Temperature | 92.300 | <.001 | 0.483 |
|  | Temperature × Gender | 0.322 | 0.572 | 0.003 |

**Appendix Table B.** Results of univariate analyses of covariance investigating the effects of time on taste ratings in Experiment 2a. The design included temperature (warm and cold) and time (pre-tasting and post-tasting) as within-participants factors and gender and thermal comfort as covariates.

| **Rating type** | ***F*** | ***p-value*** | **np2** |
| --- | --- | --- | --- |
| Buying intention | 11.40 | .002 | 0.26 |
| Tastiness | 11.33 | .002 | 0.26 |
| Healthfulness | 14.10 | .001 | 0.31 |
| Sweetness | 3.68 | .064 | 0.10 |
| Sourness | 19.26 | <.001 | 0.38 |
| Saltiness | 16.70 | <.001 | 0.34 |
| Bitterness | 11.70 | .002 | 0.27 |
| Temperature | 2.43 | .129 | 0.07 |
| Freshness | 0.48 | .493 | 0.02 |

**Appendix Table C.** Results of univariate analyses of covariance investigating the effects of time on taste ratings in Experiment 2b. The design included temperature (warm and cold) and time (pre-tasting and post-tasting) as within-participants factors and gender and thermal comfort as covariates.

| **Rating type** | ***F*** | ***p-value*** | **np2** |
| --- | --- | --- | --- |
| Buying intention | 15.94 | .001 | 0.29 |
| Tastiness | 18.20 | <.001 | 0.37 |
| Healthfulness | 8.70 | .006 | 0.22 |
| Sweetness | 8.71 | .006 | 0.22 |
| Sourness | 3.19 | .084 | 0.09 |
| Saltiness | 0.16 | .688 | 0.01 |
| Bitterness | 2.59 | .118 | 0.08 |
| Temperature | 15.57 | <.001 | 0.33 |
| Freshness | 13.71 | .001 | 0.31 |
